# circFOXM1 promotes proliferation of non-small cell lung carcinoma cells by acting as a ceRNA to upregulate FAM83D

**DOI:** 10.1186/s13046-020-01555-5

**Published:** 2020-03-30

**Authors:** Chengtao Yu, Zhuoan Cheng, Shaohua Cui, Xiaowei Mao, Botai Li, Yujie Fu, Hui Wang, Haojie Jin, Qing Ye, Xiaojing Zhao, Liyan Jiang, Wenxin Qin

**Affiliations:** 1 State Key Laboratory of Oncogenes and Related Genes, Shanghai Cancer Institute, Renji Hospital, Shanghai Jiao Tong University School of Biomedical Engineering, Shanghai, 200032 China; 2 Department of Respiratory Medicine, Shanghai Chest Hospital, Shanghai Jiao Tong University, Shanghai, 200030 China; 3 Department of Thoracic Surgery, Renji Hospital, Shanghai Jiao Tong University School of Medicine, Shanghai, 200032 China; 4 Shanghai Cancer Institute, Renji Hospital, Shanghai Jiao Tong University School of Medicine, Shanghai, 200032 China

**Keywords:** circFOXM1, Non-small cell lung cancer, miR-614, FAM83D

## Abstract

**Background:**

Biological role and clinical significance of circular RNAs (circRNAs) remain largely unknown. Herein, we aimed to investigate biological function, molecular mechanism, and clinical significance of a circular RNA FOXM1 (circFOXM1) in non-small cell lung cancer (NSCLC).

**Methods:**

Expression of circFOXM1 was measured in 48 paired samples of NSCLC by qRT-PCR. Functional roles of circFOXM1 on tumor cells were explored by in vitro and in vivo assays. Transcriptome sequencing was employed to screen the molecules involved in circFOXM1 regulatory network. RNA immunoprecipitation, luciferase analysis, RNA pull-down, and rescue assay were used to investigate potential mechanisms of circFOXM1.

**Results:**

We found that circFOXM1 was significantly upregulated in NSCLC tissues, and its upregulation was positively correlated with advanced clinical stage and poor prognosis of NSCLC patients. Gain or loss-of-function assay showed that circFOXM1 promoted cell proliferation and cell cycle progression. In vivo assays showed that silencing circFOXM1 inhibited xenograft tumor growth. Mechanically, transcriptome sequencing data indicated that silencing circFOXM1 led to the downregulation of cell cycle-related mRNAs. RNA pull-down and dual-luciferase reporter assay suggested that circFOXM1 could bind to miR-614, and FAM83D was an essential gene involved in the circFOXM1/miR-614 regulatory network.

**Conclusions:**

circFOXM1promotes NSCLC progression by interacting with miR-614 and thus inactivating the function of miR-614, which will further release the suppression of FAM83D. circFOXM1/miR-614/FAM83D regulatory network may serve as a potential therapeutic target for NSCLC patients.

## Background

Lung cancer is the leading cause of cancer-related deaths worldwide. Non-small cell lung cancer (NSCLC) is primary histological subtype and accounts for ~ 85% of lung cancers, including two major histological types, lung adenocarcinoma and lung squamous cell carcinoma [1, 2]. Recently, although the treatment of advanced NSCLC has made significant progress by targeted therapies and immunotherapies, five-year survival rate of NSCLC patients is still less than 15% [3]. One of the major challenges is to find genes essential for NSCLC progression and understand the mechanisms of them [4, 5]. In the past few decades, the investigation into the NSCLC has typically focused on protein-coding genes. However, the limitation of NSCLC therapy is still not fully overcome. Considering that the protein-coding gene accounts for only about 2% of the whole transcriptome and the remaining 98% is transcribed into non-coding RNAs (ncRNAs) [6], it is critical to examine the potential of ncRNAs and comprehend the mechanisms underlying the progression of NSCLC.

Circular RNAs (circRNAs) constitute a novel class of ncRNAs. Pervasive expression of circRNAs is a recently discovered feature in various kinds of eukaryotes, including human cells [7, 8]. circRNAs are usually considered as a master regulator of cellular processes due to their unique properties, such as abundant, stable, cell- and tissue specific expressions [9]. An increasing number of studies have revealed that circRNAs are correlated with clinical features of NSCLC patients and play as regulators in NSCLC. For example, circTP63 is positively correlated with tumor size and TNM stage, exerting oncogenic potential by sponging to miR-873 in lung squamous cell carcinoma [10]. Another circRNA, circPRKCI, also plays as an oncogene, and its therapeutic potential is confirmed in the nude mouse xenograft model and patient-derived tumor xenografts model. circPRKCI acts as a sponge for both miR-545 and miR-589 and abolishes their suppression on the oncogene E2F7 [11]. However, only a small fraction of circRNAs in the genome has been investigated until now, thus further insight into molecular mechanisms and clinical relevance of more functional circRNAs may facilitate the identification of potential biomarkers and therapeutic targets for NSCLC.

Family with sequence similarity 83-member D (FAM83D), a microtubule-associated protein, has been implicated as an oncogene in the various kinds of malignancy. Wang et al. have shown that both of the mRNA and protein levels of FAM83D are upregulated in gastric cancer, and high expression of FAM83D predicts worse overall survival and disease-free survival [12]. Liao et al. report that FAM83D is elevated in hepatocellular carcinoma, and the upregulation of FAM83D significantly promotes cell proliferation and invasion [13]. Furthermore, Yan et al. and He et al. demonstrate that FAM83D is regulated by miR-495 and miR-210 [14, 15]. Cyclin D1 (CCND1) and cyclin E1 (CCNE1) are two essential downstream mediators of FAM83D [16].

In this study, we explored the role of circFOXM1(has_circ_0025039), a circular RNA obviously upregulated in our previous microarray results (GSE126533). We first identified the circular form of circFOXM1 and then verified the upregulated expression in NSCLC tissues compared to adjacent normal tissues. circFOXM1 was correlated with advanced clinical stage and worse overall survival in NSCLC patients. In vitro and in vivo assays showed that circFOXM1 promoted cell proliferation and cell cycle progression. RNA-sequencing results suggested that cell-cycle related genes were modulated by circFOXM1. circFOXM1 functioned as a ceRNA to sponge miR-614 and upregulated FAM83D level. circFOXM1/miR-614/FAM83D regulatory network may play essential roles in cell growth of NSCLC.

## Materials and methods

### Tissues and cell lines

48 paired samples of tumor tissues(T) and their corresponding nontumor tissues(N) from patients with NSCLC were obtained from the Shanghai Chest Hospital, Shanghai Jiao Tong University (Shanghai, China). The NSCLC tissues were immediately stored in liquid nitrogen when they were removed from patients in surgery. The tissues were transported to sample bank of Shanghai Chest hospital and stored in liquid nitrogen. Subsequently, RNAs were extracted from tissues and only qualified RNAs were retained for study. An apart of qualified RNAs was used as template for cDNA reverse transcription. The RNAs and cDNAs were divided into different tubes (RNase-free and DNase-free) to avoid freeze and thaw repeatedly. The qualified RNAs and cDNAs were stored at − 80 °C until use. These samples were identified by two pathologists independently. The detailed clinicopathological features were described in Table 1. All tissues were collected from July 2013 to September 2014. Written consent approving the usage of tissues in our study was obtained from each patient. This study was approved by the Ethics Committees of the Shanghai Chest Hospital, Shanghai Jiao Tong University. Human NSCLC cell lines, including H1299, H2170, A549, and H1703, as well as BEAS-2B were purchased from the American Type Culture Collection (ATCC) and were tested negative for mycoplasma contamination. H1299, H2170, A549, and H1703 cells were cultured in RPMI 1640 medium (Life Technologies). BEAS-2B cells were cultured in MEM medium (Life Technologies). All medium was supplemented with 10% fetal bovine serum and 100 units/mL penicillin-streptomycin (Life Technologies). All cells were maintained in humidified incubator at 37 °C in a CO_2_ incubator.

**Table 1:** Correlations between circFOXM1 and clinicopathological parameters of NSCLC tissues

Clinicopathological parameters	No. of cases	Relative expression		***P*** value
		High	Low	
**Gender**				
male	41	22	19	0.219
female	7	2	5	
**Age**				
≥ 60	33	17	16	0.755
<60	15	7	8	
**Tumor size**				
≥ 4	25	18	7	0.001**
<4	23	6	17	
**Lymphatic metastasis**				
positive	27	15	12	0.382
negative	21	9	12	
**History type**				
adenocarcinoma	15	6	9	0.459
squamous	33	16	17	
**TNM stage**				
I/II	25	9	16	0.043*
III/IV	23	15	8	

* *P* < 0.05,** *P* < 0.01

### RNA and DNA extraction

Total RNAs of tissues and cells were extracted by using Trizol reagent (Invitrogen). All experiment operations were followed the manufacturer’s instruction of Trizol reagent. The procedure of RNAs extracted from nuclear fractions or cytoplasmic fractions were according to PARIS Kit (Life Technologies) manufacturer’s protocol. For DNA extraction, cells were rinsed with PBS twice and then extracted by Genomic DNA Isolation Kit (Sangon Biotech, China).

### RNase R treatment

RNase R (Epicentre Technologies) was used to treat with total RNAs. Briefly, extracted RNAs aliquots from H1299 and H2170 cells were split into two parts: one for RNase R digestion and another for control with digestion buffer only. For RNase R digestion, 2 μg of total RNA was mixed with 2 μl 10 × RNase R Reaction Buffer and 2 μl RNase R (20 U/μl); for control, RNase R was replaced with DEPC-treated water. Then, the RNA samples were incubated at 37 °C water bath heater for 30 min. The detection of circFOXM1 and FOXM1 mRNA was analyzed by PCR, RT-PCR or qRT-PCR. RNase R treated RNA was used only for detecting resistance of circFOXM1 to RNase R exonuclease digestion. All primers were listed in Additional file 1: Table S1.

### Reverse transcription PCR(RT-PCR) and quantitative real-time PCR (qRT-PCR)

For RT-PCR, 500 ng RNA was treated with gDNA wiper for 2 min at 42 °C and then was used to synthesize cDNA by using Hiscript Revert 1st First Strand cDNA Synthesis Kit (Vazyme, China). cDNA was used as templates to amplify by DNA Polymerase (Life Technologies), and products were further verified by using 1.5% agarose gel electrophoresis.

For qRT-PCR, only the cDNA was used as template and qRT-PCR assays were investigated by AceQ qPCR SYBR Green Master Mix (Vazyme, China) kits on ABI 7500 qPCR system. The circRNA and mRNA levels were normalized by β-actin. miRNA level was normalized by U6. The relative expression levels were determined by the 2^−ΔCt^ or 2^−ΔΔCt^ method. To determine the absolute quantity of RNA, the purified PCR product amplified from cDNA corresponding to the circFOXM1 and FAM83D sequence was serially diluted to generate a standard curve, respectively. Briefly, circFOXM1 and FAM83D form cDNAs were amplified, purified and measured. Then they were serially diluted to be as templates for qRT-PCR. The standard curves were drawn according to the Ct values at different concentrations. According to the standard curves, copy numbers of circFOXM1 and FAM83D in NSCLC cell lines were calculated.

### Plasmid construction and transfection

To construct circFOXM1 ectopic overexpression plasmid, the sequences of exon 4 and exon 5 in FOXM1(amplified from cDNAs of H1299 cells) were cloned into pZW-circRNA vector (a gift from Ling-Ling Chen Lab) [17]. To construct circFOXM1 knockdown plasmids (sh-circFOXM1), fragments targeting the circFOXM1 junction sites were cloned into pGreenPuro vector (System Biosciences). Likely, fragments targeting FAM83D mRNAs were constructed into pGreenPuro vector to generate FAM83D knockdown plasmids (sh-FAM83D). For dual-luciferase assay, wild-type and mutant fragments of circFOXM1 as well as FAM83D 3′ UTR were cloned into pmirGLO vector (Promega) to form luciferase reporter vector. The sequences of primers were listed in Additional file 1: Table S1.

For pZW-circFOXM1, sh-circFOXM1 or sh-FAM83D transfection, 2 × 10^5^ cells were seed in 60 mm dishes for 24 h before transfection. For shRNA-circFOXM1 or shRNA-FAM83D stable cell line construction, 1 × 10^5^ cells were seed in 60 mm dishes for 24 h before virus infection. Lentivirus was added into culture medium with polybrene, followed by selection with puromycin (2 μg/ml) for 2 weeks. For dual-luciferase assay, plasmids with wild-type or mutant fragments were co-transfected with miR-614, respectively.

### Transcriptome sequencing

Total RNAs were isolated from H1299 cells with circFOXM1silencingorcontrol by using TRIzol reagent (Invitrogen) and purified by RNeasy Mini Kit (Qiagen). Transcriptome sequencing was conducted using Illumina HiSeq™ 2000 by Sangon (Sangon Biotech, China). Differently expressed transcripts were selected by |fold change| > 2 and *FDR* < 0.1. Results were collected in Additional file 2: Table S2.

### Gene set enrichment analysis

Gene set enrichment analysis (GSEA) software (version 3.0, www.broadinstitute.org/gsea/) was employed to identify gene sets that were significantly overrepresented among genes up- or down-regulated in circFOXM1 silencing cells. Briefly, gene expression profiles were generated from circFOXM1knockdown and control cells by RNA-sequencing. GSEA v3.0 software was used to explore the distribution of members of the gene sets from the MSigDB database. In this bioinformatics analysis, if the most members in a gene set were positively or negatively correlated with the circFOXM1 expression, the set was termed associated with circFOXM1.

### CCK-8 assay

1 × 10^3^ cells were seeded in 96-well plates and were cultured for 1 ~ 5 day, followed by incubation with 10 μl of CCK-8 assay solution per well for 2 h (Dojindo Laboratories, Japan). The absorbance values at 450 nm were then assessed.

### Colony formation assay

A density of 1 × 10^3^ cells per well were incubated in 6-well plates. After 2 weeks’ incubation, a total of 1% of crystal violet (Beyotime Biotechnology, China) was applied to stain cell clones which were fixed with methanol.

### Flow cytometry analysis

After harvesting by trypsinization, cells were washed with pre-cold PBS buffer, and then fixed in 75% ice-cold ethanol. Before staining, cells were resuspended in cold PBS and subjected to digestion with 2 μg/ml RNase A at 37 °C for 30 min, followed by labeling with 15 μg/ml propidium iodide (Beyotime Biotechnology, China) for 15 min at room temperature. Cell cycle profiles of labeled-cells were analyzed using a FACS Calibur flow cytometer (BD Biosciences).

### Fluorescence in situ hybridization (FISH)

Cells were rinsed by PBS and then fixed in 4% formaldehyde for 10 min room temperature. The cells were further permeabilized in PBST (0.5% Triton X-100) on ice for 10 min, and then prehybridized with prehybridization buffer. After rinsing once in 2 × SSC, hybridization was performed using Cy3-labelled probe at 42 °C overnight. After co-staining with DAPI, the signals of the probe were captured by confocal microscopy (Zess 7100).

### RNA pull-down assay

For miR-614 pull-down assay, the 3’end biotinylatedmiR-614 mimics or miRNA control (Sangon, China) were transfected into cells at a final concentration of 100 nM for 48 h before harvest. Then IP Cell lysis Buffer (Sangon Biotech, China) and complete protease inhibitor cocktail (Roche Applied Science) were added into the cell pellets, and incubated on ice for 10 min. Biotin-coupled RNA complex was pulled down by incubating the cell lysates with streptavidin-coated magnetic beads (Life Technologies) by centrifugation at 10,000×g for 10 min. The abundance of targets was evaluated by qRT-PCR analysis.

For circFOXM1 pull-down assay, 1 × 10^7^cells that expressed circFOXM1 were harvested, lysed, and sonicated. The biotin-coupled probe of circFOXM1 or probe control was incubated with magnetic beads, respectively. After 2 h incubation, cell lysates were incubated with the probes overnight at 4 °C. After the incubation, the bound RNAs were washed for six times with wash buffer and purified for the qRT-PCR analysis. The biotinylated-probe was designed and synthesized by Sangon (Shanghai, China).

### Immunoblotting

Extracted protein samples were quantified and boiled in SDS/β-mercaptoethanol buffer, then loaded into polyacrylamide gels. After separation by electrophoresis, the proteins were transferred onto PVDF membranes (Millipore). The membrane was incubated with rabbit anti-FAM83D antibody (ab236882, Abcam) or anti-CCND1 antibody (26939–1-AP, proteintech) or anti-CCNE1(11554–1-AP, proteintech) or anti-FOXM1 antibody (13147–1-AP, proteintech) or anti-β-actin antibody (20536–1-AP, proteintech) at 4 °C for 12 h, followed by an incubation with secondary antibody (proteintech) for 1 h. Bands were detected by a Bio-Rad ChemiDoc XRS system.

### RNA immunoprecipitation (RIP)

RIP assays were performed by using a Magna RIP RNA-Binding Protein Immunoprecipitation Kit (Millipore) according to the protocol. Ago2 antibody was used for RIP (Cell Signaling Technology). Co-precipitated RNA was detected by qRT-PCR.

### Luciferase assay

Cells were plated onto 96-well plates and grown to 70% confluence. For circFOXM1 and miR-614 luciferase assay, the cells were co-transfected with 50 nM miR-614 mimics and 200 ng pmirGLO-circFOXM1 vector, or corresponding mutant type (mut). For FAM83D and miR-614 luciferase assay, the cells were co-transfected with 50 nM miR-614 mimics and 200 ng pmirGLO-FAM83D vector, or corresponding mutant type (mut). Dual luciferase assay kit (promega) was used for dual luciferase assay. At 48 h post-transfection, cells were collected, and Renilla luciferase activity was assessed. Results are assessed as the ratio of Firefly luciferase activity to Renilla luciferase activity.

### Xenografts experiments

Five-weeks’ old male BALB/c nude mice were chosen and randomly divided into four groups for our experiment. H2170 cells transfected with vector control, pZW-circFOXM1, sh-circFOXM1 or negative control (sh-NC) were subcutaneously injected into the back of the nude mice (1 × 10^6^, 100 μl). The volume and weight of tumors were measured for 35 days. All animal experiments were performed under approval by the Shanghai Medical Experimental Animal Care Commission.

### Immunohistochemistry

Tumor sections were subjected to deparaffinize and rehydrate with gradient ethanol solutions. After being immersed in antigen retrieval solution (AR0023, Boster, China) and heated for 15 min, sections were then incubated with anti-human Ki-67 antibody (M7240, Dako, Denmark) for 1 h and counterstained with hematoxylin (BA-4226, BASO, China) for 2 min.

### Statistical analysis

Data were expressed as the mean ± standard deviation from at least three independent experiments. Survival analysis was performed with the Kaplan-Meier method, and the log-rank test was used for comparisons. Statistical results were analyzed using Prism software (GraphPad Software). Student’s t-test was used to compare two experimental groups. A probability of 0.05 or less was considered as statistical significance.

## Results

### Identification and characteristic of circFOXM1 in NSCLC

**Fig. 1:**
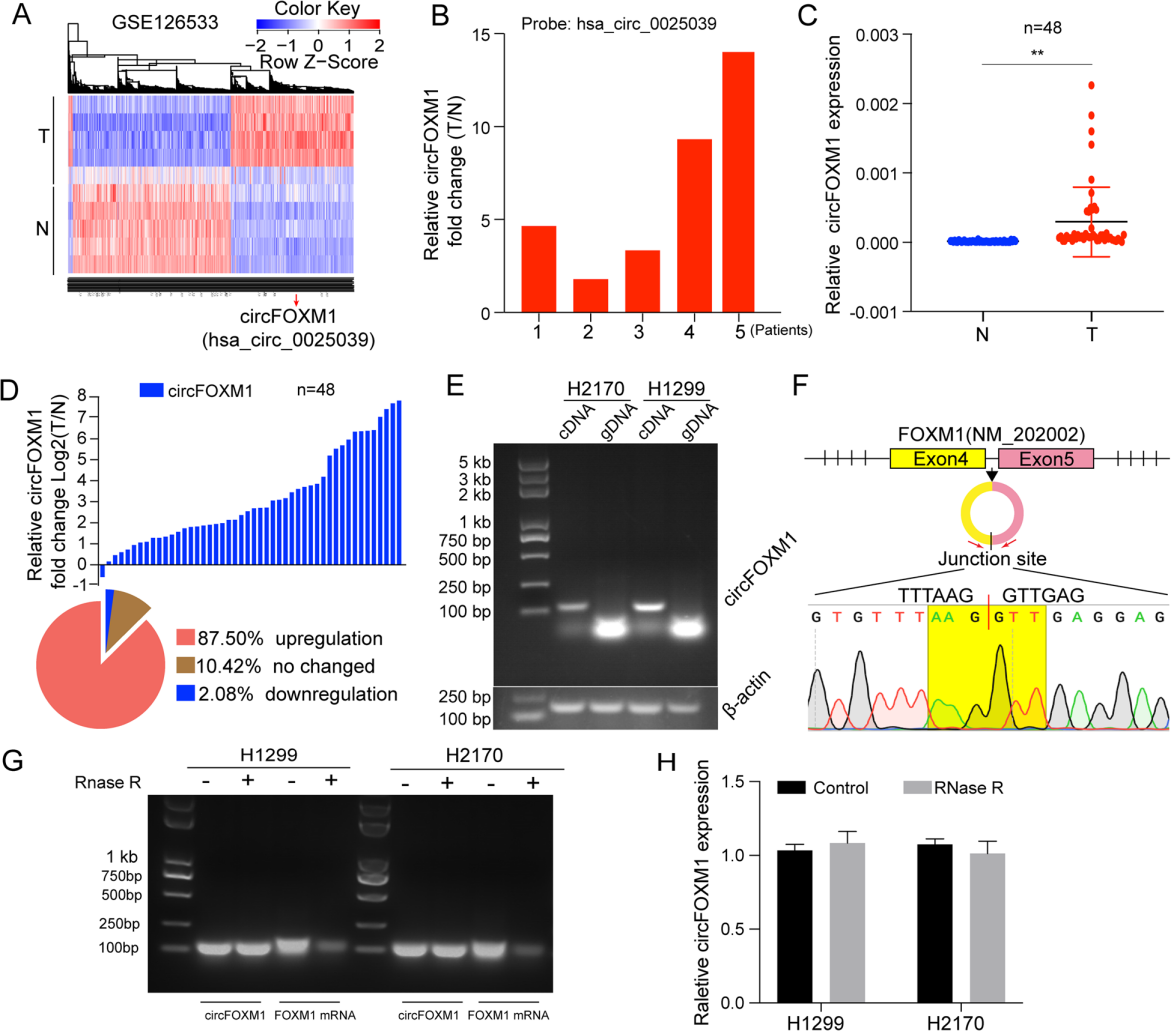
Identification and characteristic of circFOXM1 in NSCLC. **a** circFOXM1 was one of the most upregulated circRNAs based on the analysis of the5 paired samples of NSCLC tissues in GSE126533. T, tumor tissue; N, nontumor tissue. **b** Relative fold change (T/N) of circFOXM1 in GSE126533. **c** Expression of circFOXM1 was determined in 48 paired samples of NSCLC tissues. β-actin was used as control. **d** Histogram and pie chart of the proportions of NSCLC samples in which circFOXM1 expression was upregulated (42/48, 87.50%, red), downregulated (1/48, 2.08%, blue), or no change (5/48, 10.42%, brown). Log2 (T/N) expression value > 1 as higher expression, which < − 1 as lower expression, and between − 1 and 1 as no significant change. **e** PCR analysis for circFOXM1 in cDNA and genomic DNA (gDNA). **f** A schematic view of circFOXM1 genomic loci. circFOXM1 is produced at the FOXM1 gene (NM_202002) locus containing exons 4–5. The back-splice junction of circFOXM1 was identified by Sanger sequencing. **g** RT-PCR analysis for circFOXM1 and FOXM1 mRNA after treatment with RNase R in NSCLC cells. **h** qRT-PCR analysis for circFOXM1 after treatment with RNase R in NSCLC cells. **P < 0.01

Based on the analysis for our previous microarray data (GSE126533), we found that has_circ_0025039 (chr12:2975558–2,977,920) was evaluated as an upregulated circRNA in NSCLC (Fig. 1a-b). By browsing the human reference genome (GRCh37/hg19), we knew that has_circ_0025039 was derived from the exon 4 and 5 of FOXM1 loci, and thus named it circFOXM1 in this study. Subsequently, we validated the upregulation of circFOXM1 in another 48 paired samples of NSCLC by qRT-PCR. Results showed that the expression of circFOXM1 was significantly higher in 87.5% (42 of 48) of NSCLC tissues than adjacent nontumor tissues (Fig. 1c-d). Analysis for clinical relevance revealed that the expression level of circFOXM1 was more likely to be positively associated with tumor size and TNM stage (Table 1). In addition, high expression of circFOXM1may predict an unfavorable overall survival of NSCLC patients (Fig. S1A).

To verify that circFOXM1 was circular RNA rather than products of trans-splicing or genomic rearrangements, circRNA identification assays were performed. Firstly, using cDNA or genomic DNA from cells as templates, we found that circFOXM1 was only amplified from cDNA, while no specific amplification product was observed from genomic DNA (Fig. 1e). The back-spliced junction in PCR products of circFOXM1 was also identified by Sanger sequencing (Fig. 1f). Results of resistance to RNase R exonuclease digestion further confirmed that circFOXM1 was circular in form (Fig. 1g-h). Taken together, these results indicate that circular RNA circFOXM1 is upregulated in NSCLC, and high expression of circFOXM1 is associated with advanced stage and poor survival of NSCLC patients.

### circFOXM1 promotes cell proliferation and tumor growth

Next, we measured the expression of circFOXM1in NSCLC cell lines by qRT-PCR. Results showed that circFOXM1 was highly expressed in NSCLC cell lines compared to human normal bronchial epithelium cells (BEAS-2B) (Fig. S1B). For constructing ectopic circFOXM1 overexpressing vector, exons 4 and 5 of FOXM1 were cloned into the pZW vectors (Fig. S1C). For silencing circFOXM1, we constructed the short hairpin RNA vector, which specifically targeted the back-splicing junction of circFOXM1(Fig. S1D). As expected, results showed that circFOXM1 was successfully expressed more than 40-fold changes by pZW-circFOXM1 than controls in H1299 and H2170 cells, while FOXM1 mRNA expression had no obvious change (Fig. S1E). Likely, sh-circFOXM1 could successfully decrease the expression of circFOXM1 by about two-fold changes but had no effect on the expression of FOXM1 mRNA (Fig. S1F). Besides, we also found that circFOXM1 had no effect on the level of FOXM1 protein (Fig. S1G-H).

**Fig. 2:**
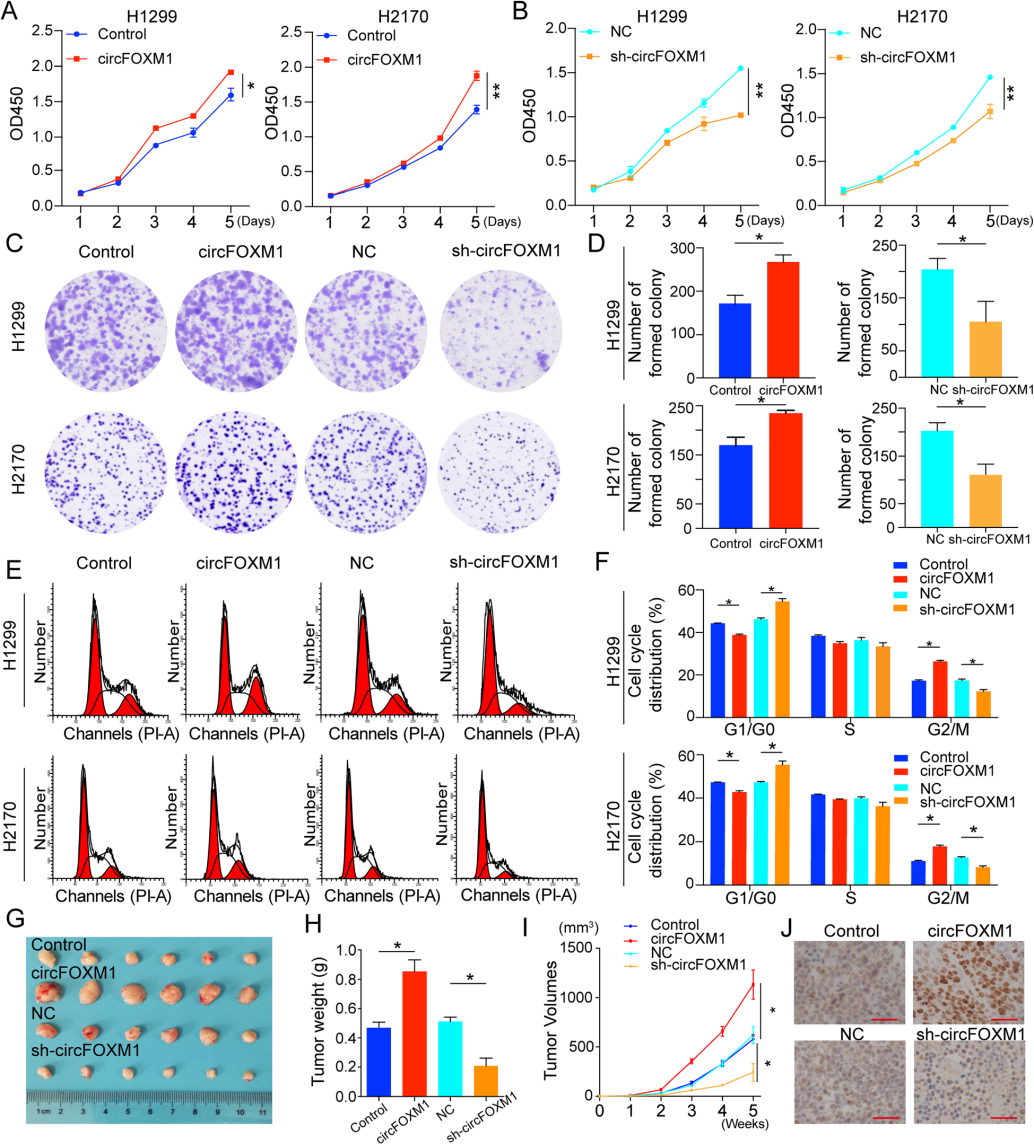
circFOXM1 promotes cell proliferation and tumor growth. **a-b** CCK-8 assay was performed to determine the viability in H1299 and H2170 cells with overexpressing or silencing circFOXM1. **c-d** Colony formation assay was employed to examine the colony formation abilities of H1299 and H2170 cells with overexpressing or silencingcircFOXM1. **e-f** Flow cytometry analysis showed an alteration in the proportion of cell in G1 phase and G2/M phase in H1299 and H2170 cells with overexpressing or silencing circFOXM1. **g-i** The volume and weight of subcutaneous xenograft tumors of H2170 cells isolated from nude mice (*n* = 6 mice per group). **j** Immunohistochemical staining for cell proliferation markers Ki-67 in subcutaneous xenograft tumors. *P < 0.05; **P < 0.01

Subsequently, in vitro assays were performed to investigate potential roles of circFOXM1. CCK-8 results revealed that the viability of both H1299 and H2170 cells was increased in circFOXM1 overexpressing group while decreased in circFOXM1 silencing group (Fig. 2a-b). Colony formation assays showed that the ability of colony formation was significantly improved in circFOXM1 overexpressing group, and reduced in circFOXM1 silencing group (Fig. 2c-d). Furthermore, cell cycle analyses showed that the number of cells was increased in G2/M phase when overexpressing circFOXM1. In contrast, silencing circFOXM1 made cells arrested in G0/G1 phase (Fig. 2e-f). These data indicate that circFOXM1 promotes NSCLC cell proliferation and cell cycle progression in vitro.

To identify the effect of circFOXM1 on tumor growth in vivo, we established a nude mice xenograft model by implanting H2170 cells with circFOXM1overexpressing or circFOXM1 silencing. Results showed that tumor cells with circFOXM1overexpressing grew rapidly compared to control. In addition, Ki-67 staining showed that circFOXM1 made more Ki-67 positive cells. In contrast, silencing circFOXM1 obviously blocked upon tumor growth and decreased Ki-67 staining cells (Fig. 2g-j). Taken together, our findings suggest that circFOXM1 may play oncogenic roles in NSCLC progression.

### Silencing circFOXM1 influences the expression of cell cycle-related genes

**Fig. 3:**
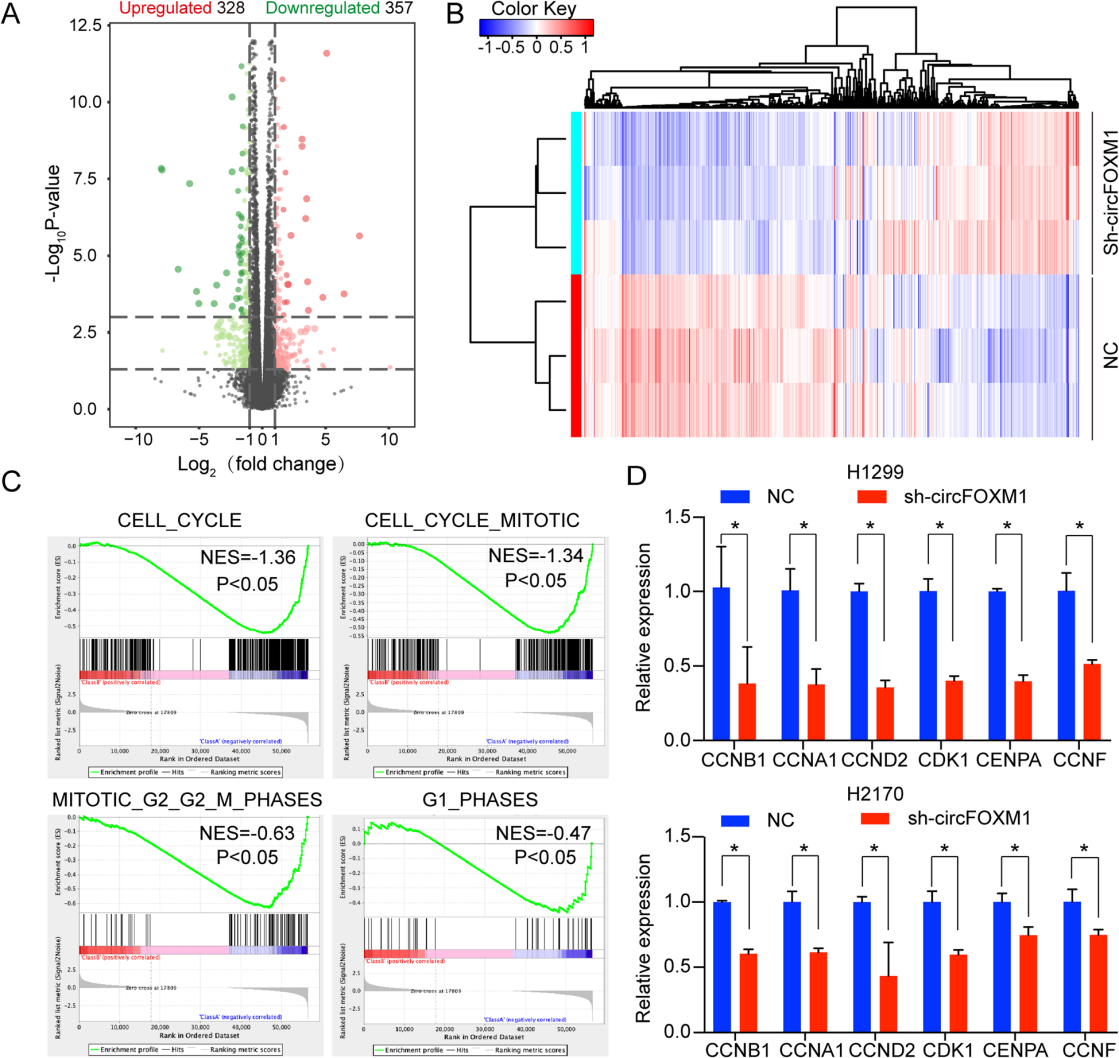
Silencing circFOXM1 influences expression of cell cycle-related genes. **a** A volcano plot illustrated 685 differentially expressed genes in H1299 cells after silencing circFOXM1 by RNA sequencing analysis (|Fold change| > 2, FDR < 0.1). **b** A cluster heat map showed all 685 differentially expressed genes in H1299 cells after silencing circFOXM1. **c** Gene Set Enrichment Analysis (GSEA) indicated that the signatures of cell_cycle, cell_cycle_mitotic, mitotic G2_G2_M phase, and G1 phase were significantly enriched in circFOXM1 silencing group compared to control. NES: Normalized Enrichment Scores. **d** Validation of 6 cell cycle-related genes by qRT-PCR in H1299 and H2170 cells with circFOXM1 silencing. *P < 0.05

Using RNA-sequencing technology, we identified 685 dysregulated genes (|Fold change| > 2, FDR < 0.1) (328 genes upregulated and 357 genes downregulated) after silencing circFOXM1in H1299 cells (Fig. 3a, Additional file 2: Table S2). Hierarchical clustering heat map showed different clustering patterns between circFOXM1 silencing group and control group (Fig. 3b). To further illustrate the mechanism of circFOXM1, we analyzed GSEA results and found that the signatures of cell cycle, cell cycle mitotic, mitotic G2/G2-M phase, and G1 phase were significantly enriched in downregulated genes, indicating that these biologic processes may be suppressed by circFOXM1 silencing (Fig. 3c). Moreover, we tested and verified six genes of these signatures by qRT-PCR. Results showed that the expression of CCNB1, CCNA1, CCND2, CDK1, CENPA, and CCNF were downregulated in both H1299 and H2170 cells with circFOXM1 silencing (Fig. 3d). These results were consistent with above phenotypes that silencing circFOXM1 made cell cycle arrested in G0/G1 phase. Thus, circFOXM1 may promote cell proliferation by modulating cell cycle-related genes.

### circFOXM1 facilitates cell proliferation by sponging miR-614

**Fig. 4:**
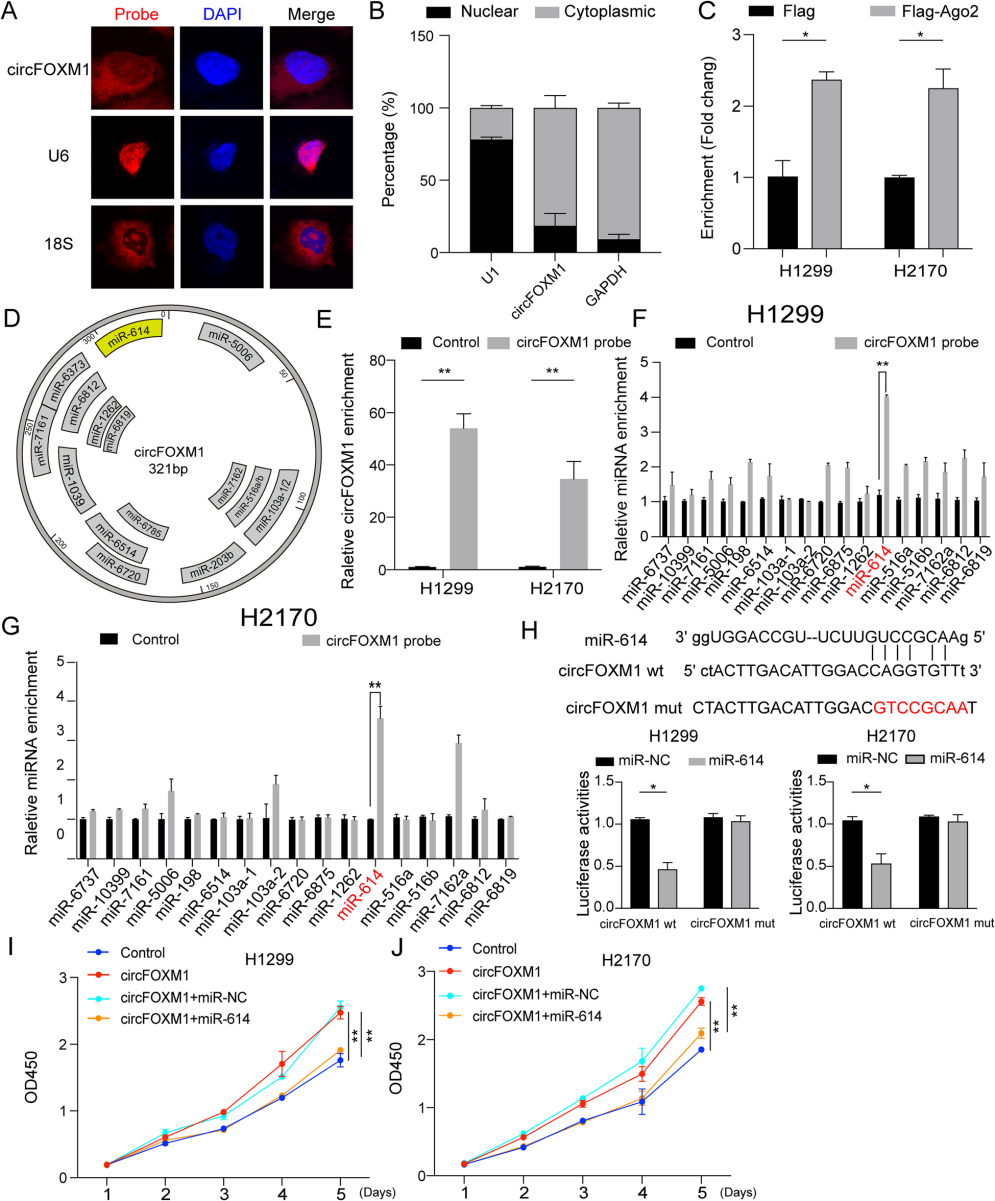
circFOXM1 facilitates cell proliferation by sponging miR-614. **a** RNA fluorescence in situ hybridization (FISH) showed that circFOXM1 was predominantly localized in cytoplasm in H1299 cells. U6 was mainly localized in nucleus. 18S was mainly localized in cytoplasm. **b** Cytoplasmic and nuclear RNA fractionation experiments showed that circFOXM1 was mainly located in the cytoplasm in H1299 cells. **c** Ago2 RIP assay was used for the detection of circFOXM1 in H1299 and H2170 cells expressing Flag-Ago2 or Flag-tag. **d** The putative binding sites of 17 predicted miRNAs on circFOXM1. **e** RNA pull-down assay was used for the detection of circFOXM1 by circFOXM1 specific probe. **f-g** Relative expression of circFOXM1 putative binding miRNAs was examined by qRT-PCR analysis by circFOXM1 specific probe. **h** Luciferase activity of luc-circFOXM1 wt or Luc-circFOXM1 mut in H1299 and H2170 cells co-transfected with miR-614 mimics. **i-j** Cell proliferation analysis of H1299 and H2170 cells transfected with control, circFOXM1, circFOXM1 + miR-NC (miRNA control), or circFOXM1 + miR-614. *P < 0.05; **P < 0.01

Many studies have revealed that circRNAs play as the major regulators through miRNA sponge, acting as ceRNAs by blocking Ago2-mediated silencing complex [18, 19]. To investigate whether circFOXM1 could function as a ceRNA, we first detected its subcellular localization. As results shown in FISH assay and nuclear/cytoplasmic fraction assay, the majority of circFOXM1 was preferentially localized in the cytoplasm (Fig. 4a-b). RNA-protein immunoprecipitation results revealed that circFOXM1 could bind Ago2 protein (Fig. 4c, Fig. S2A). Thus, we supposed that circFOXM1 had the potential to function as a ceRNA. Then, we determined to screen potential miRNAs binding to circFOXM1 by miRanda prediction (http://159.226.67.237:8080/new/analysis_mirna.php), and found that 17 miRNAs (Energy<− 23) were predicted as binding partners with circFOXM1 (Fig. 4d). RNA pull-down assays showed that specific probe against circFOXM1 could enrich circFOXM1 RNAs more than 30-fold changes compared with control (Fig. 4e), and miR-614 was the most enriched miRNA in the captured fraction of circFOXM1 specific probe compared with control in both H2170 and H1299 cells (Fig. 4f-g). To further validate the interaction between circFOXM1 and miR-614, we performed luciferase assays containing wild-type and mutated putative binding sites of circFOXM1 to verify the direct interaction. Our results showed that the luciferase activities of circFOXM1 wild-type reporter were significantly reduced when transfected with miR-614 mimics compared to control or circFOXM1 mutated luciferase reporter. Importantly, cells transfected with plasmids of mutant circFOXM1 could not significantly promote cell proliferation and cell cycle progression (Fig. S2B-C). We then examined the function of miR-614 by several methods, including CCK-8 assay, colony formation assay, and cell cycle assay. The results of these assays showed that miR-614 inhibited cell proliferation and cell cycle progression (Fig. S3A-D). Moreover, we also found that miR-614 was downregulated in NSCLC tissues by analyses for our microarray data and qRT-PCR results (Fig. S3E-F). Overall survival analysis in Kaplan-Meier Plotter database [20] (https://kmplot.com) showed that high expression of miR-614 predicted good prognosis in NSCLC (Fig. S3G). Furthermore, CCK-8 assays revealed that miR-614 could weaken the promotion effect of circFOXM1 in both H1299 and H2170 cells (Fig. 4i-j). Taken together, these results indicate that circFOXM1 promotes cell proliferation mediated by miR-614.

### miR-614 inhibited cell proliferation via FAM83D

**Fig. 5:**
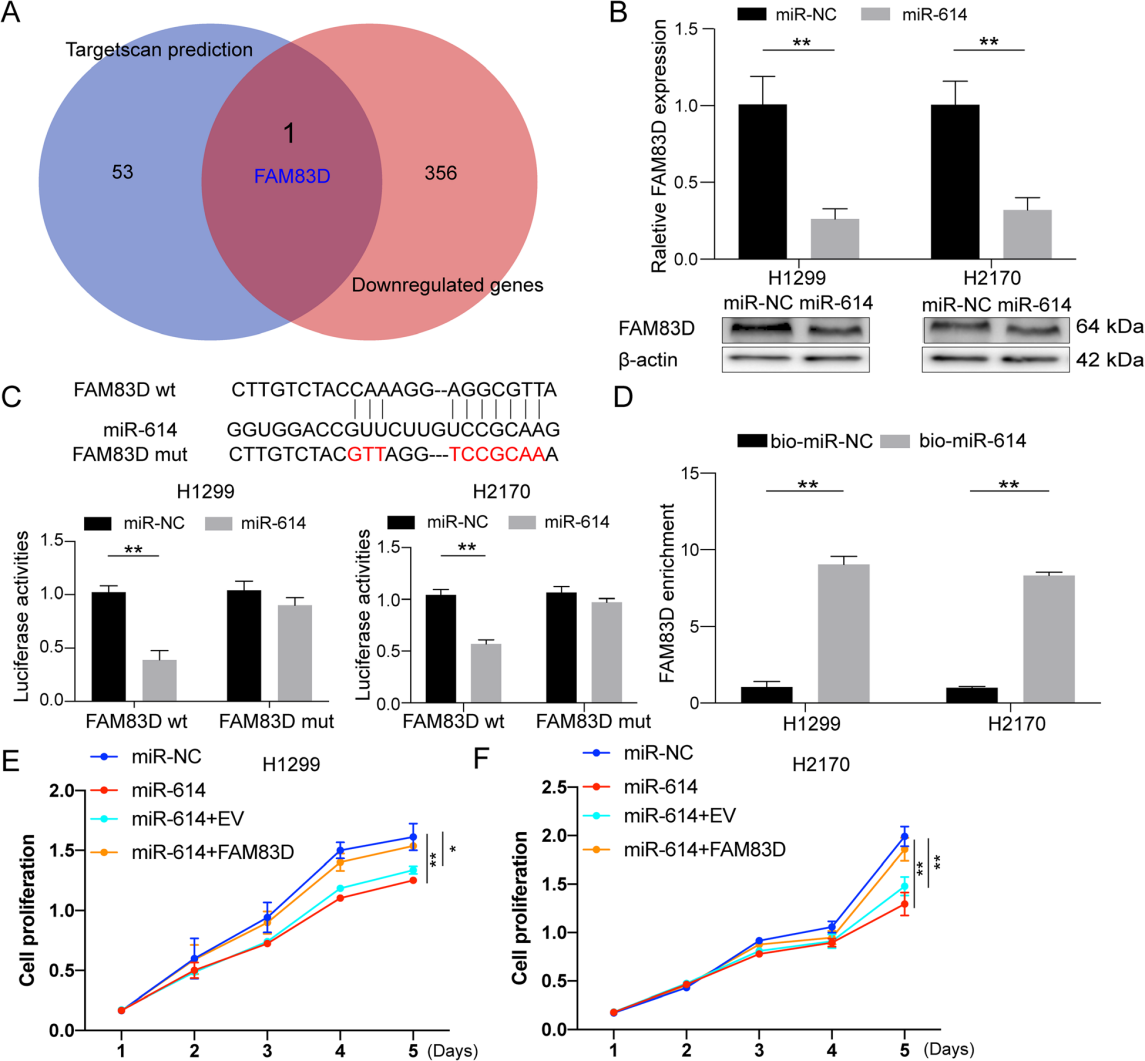
miR-614 inhibited cell proliferation via FAM83D. **a** Venn diagram showed the intersection between miR-614 potential targets predicted by Targetscan (blue circle, 8mer sites) and downregulated genes (red circle, |fold change| > 2, FDR < 0.1) after silencing circFOXM1 in H1299 cells. FAM83D is the only gene in the overlapping of the two datasets. **b** mRNA and protein levels of FAM83D after transfection of miR-614 or miR-NC in H1299 and H2170 cells. **c** Upper panel: a schematic illustration showed the putative binding sites of the miRNAs associated with 3’UTR of FAM83D. Lower panel: luciferase activity of 3′ UTR of Luc-FAM83D in H1299 and H2170 cells transfected with miR-614 mimics or miR-NC. **d** RNA pull-down assay was used for the detection of FAM83D with either miR-614 or miR-NC probe in H1299 and H2170 cells. **e-f** Cell proliferation analysis of H1299 and H2170 cells transfected with miR-NC, miR-614, miR-614 + empty vector (EV), or miR-614 + FAM83D by CCK-8 assay. *P < 0.05; **P < 0.01

According to ceRNAs theory [19], circFOXM1 should share the miR-614 with mRNA and thus released the repression of mRNA by competitively binding with miR-614. Next, we first predicted the targets of miR-614 by Targetscan software(http://www.targetscan.org/vert_72/) [21]. The prediction results showed that 54 mRNAs had the potential to be regulated by miR-614(Additional file 3: Table S3). Then, we analyzed the downregulated RNAs in our RNA-sequencing data after silencing circFOXM1(Additional file 2: Table S2). Only FAM83D was a common target of miR-614 between prediction targets of Targetscan software and downregulated RNAs after silencing circFOXM1(Fig. 5a).Results of western blot and qRT-PCR showed that the protein and mRNA levels of FAM83D were downregulated by miR-614 transfection(Fig. 5b). Luciferase assays containing wild-type and mutated putative binding sites of FAM83D 3’UTR were performed to verify the direct interaction between them. Results showed that the luciferase activities of FAM83D wild-type reporter were significantly reduced when transfected with miR-614 mimics compared to control reporter or FAM83D 3’UTR mutated luciferase reporter (Fig. 5c). RNA pull-down results suggested that miR-614 could enrich FAM83D more than five-fold changes (Fig. 5d). These results indicate that FAM83D is a direct target of miR-614. CCK-8 assays, colony formation assays, and cell cycle assays showed that silencing FAM83D inhibited cell proliferation, colony formation, and cell cycle progression (Fig. S4A-D). In addition, CCK-8 assays showed that overexpression of FAM83D could rescue the inhibitory effects of miR-614 (Fig. 5e-f). We also found that FAM83D was frequently upregulated in NSCLC patients, and high expression of FAM83D predicted poor prognosis [20](Fig. S4E-G). Taken together, these results suggest that FAM83D is a target of miR-614.

### circFOXM1 promotes cell proliferation via the miR-614/FAM83D axis

**Fig. 6:**
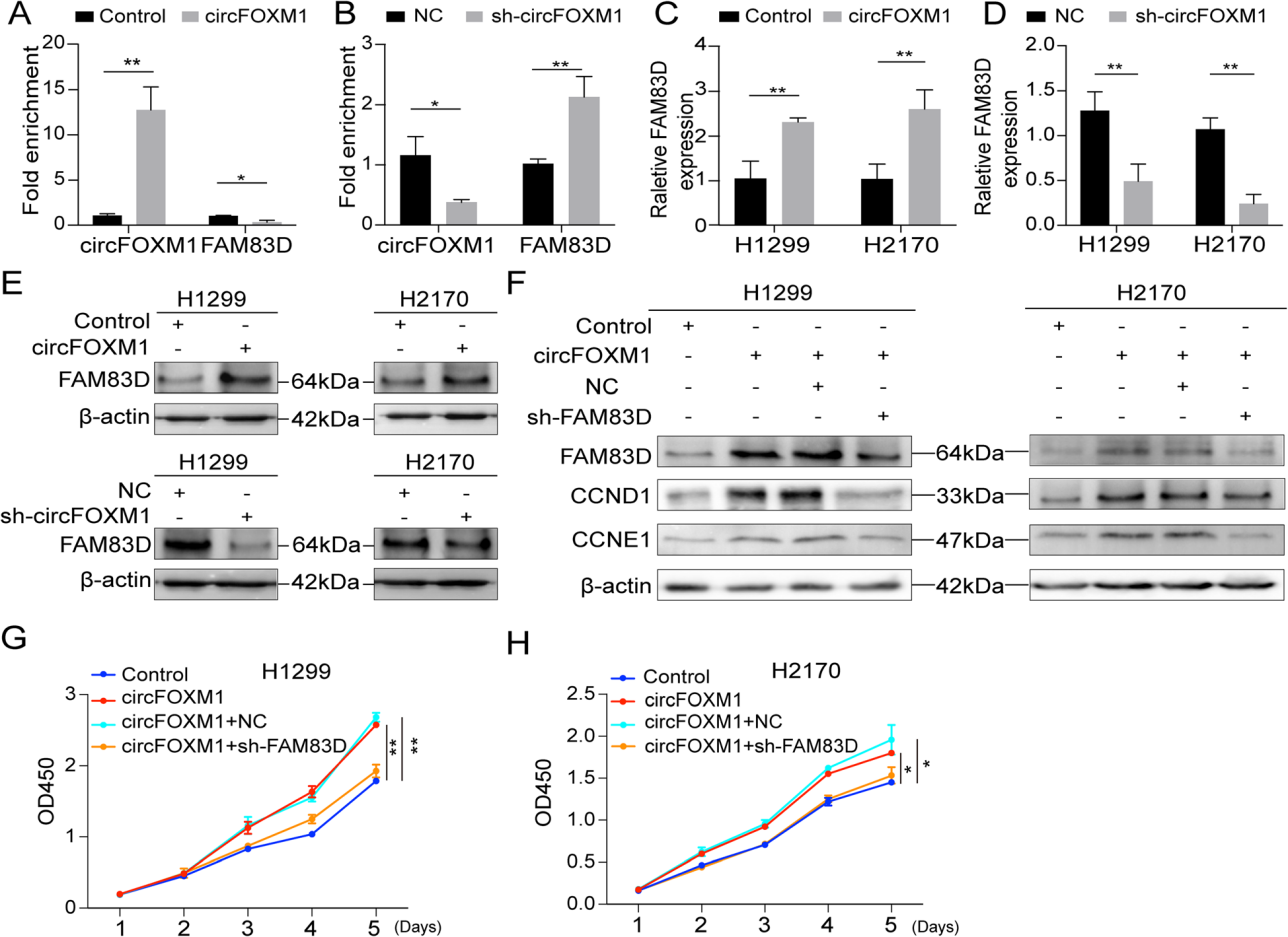
circFOXM1 promotes cell proliferation via the miR-614/FAM83D axis. **a-b** RNA pull-down assay was used for the detection of circFOXM1 and FAM83D by 3′-end biotinylated miR-614. **c-d** qRT-PCR analysis for mRNA levels of FAM83D in NSCLC cells with circFOXM1 overexpressing or silencing. **e** Western blot analysis for protein levels of FAM83D in NSCLC cells with circFOXM1 overexpressing or silencing. **f** Western blot analysis for downstream proteins of FAM83D after transfection with control, circFOXM1, circFOXM1 + NC, or circFOXM1 + sh-FAM83D. **g-h** Cell proliferation analysis for NSCLC cells transfected with control, circFOXM1, circFOXM1 + NC, or circFOXM1 + sh-FAM83D by CCK-8 assay. *P < 0.05; **P < 0.01

qRT-PCR results showed that the copies of circFOXM1 was about 160 and 220 in H1299 and H2170 cells (per 20 pg RNA), while FAM83D were about 220 and 750 in H1299 and H2170 cells (per 20 pg RNA), respectively. These data suggested that expression levels of circFOXM1 and FAM83D were near (Fig. S5). RNA pull-down assays showed that overexpression of circFOXM1 led to less enrichment of FAM83D in miR-614 pull-down assay. However, silencing circFOXM1 caused a significant increase in the recruitment of FAM83D in miR-614 pull-down assay (Fig. 6a-b). Furthermore, results of western blot and qRT-PCR showed that overexpression of circFOXM1 upregulated the mRNA and protein levels of FAM83D, while silencing circFOXM1 had the opposite effects (Fig. 6c-e). In addition, circFOXM1 expression was positively correlated with FAM83D in clinical NSCLC tissues (*r* = 0.466, *P* = 0.0008) (Fig. S4H). These evidences indicate that circFOXM1 can function as a miR-614 sponge to promote FAM83D expression.

It has been reported that FAM83D can regulate proteins to affect cell proliferation and cell cycle progressions, such as CCND1 and CCNE1 [16]. As showed in Fig. 6f, overexpression ofcircFOXM1 increased protein levels of CCND1 and CCNE1while silencing FAM83D suppressed the effects of circFOXM1 on upregulation of CCND1 and CCNE1, and their expression levels were decreased. CCK-8 assays also showed that silencing FAM83D decreased the promotion effects of circFOXM1 on cell proliferation (Fig. 6g-h). These results indicate that circFOXM1 promotes cell proliferation of NSCLC cells via the miR-614/FAM83D regulatory axis.

## Discussion

circRNAs are first observed in the 1970s [7]. In the initial studies, the evidence of circRNAs is provided in the viral genetic materials [22–24]. Then, many genes are reported to produce individual circRNAs, such as ETS-1, SRY, and P450 in different cells. However, circRNAs are still considered as rare events with unclear biological functions [25–28]. Until 2012, with the development of high-throughput RNA sequencing and bioinformatics analyses, the landscape of human circRNAs has been disclosed in different cells and tissues [6, 29]. Since then, the focus of circRNA research has shifted to elucidate the association with human diseases, including cancers. Serval studies have been reported that the abnormally expressed circRNAs are correlated with clinical features and have the potential to be biomarkers in NSCLC [9, 30, 31]. In this study, we found a circular RNA, has_circ_0025039 derived from the exons 4 and 5 of FOXM1 (named circFOXM1), was obviously upregulated in NSCLC tissues. High expression of circFOXM1 positively correlated with tumor size and advanced stage, and predicted poor prognosis for NSCLC patients.

To date, A large number of studies report that many genes, previously considered as protein-coding genes, can produce circRNAs by back-splicing [32, 33]. Interestingly, it has been described that these circRNAs can be similar or completely different from their maternal gene functions [34–36]. In this study, functional assays showed that circFOXM1 played as oncogenic roles in NSCLC, which is similar to function of its parent gene FOXM1. Liu et al. report that hsa_circ_0025033, another circular RNA derived from the exons 2–10 of FOXM1, also promotes the NSCLC progression [37]. Nowadays, a total of 14 FOXM1-derived circRNAs have been recorded in the circbase database (http://www.circbase.org/) including hsa_circ_0025033, hsa_circ_0025031, hsa_circ_0025035, hsa_circ_0025032, hsa_circ_0025041, hsa_circ_0025039 (circFOXM1), hsa_circ_0025038, hsa_circ_0025037, hsa_circ_0025042, hsa_circ_0025040, hsa_circ_0025030, hsa_circ_009831, hsa_circ_0025036, and hsa_circ_0025034. In our microarray data (GSE126533), 13 circRNAs derived from FOXM1 gene were detected except for hsa_circ_0098318. Among the 13 circRNAs, 5 circRNAs including hsa_circ_0025032, hsa_circ_0025038,hsa_circ_0025042,hsa_circ_0025031, and hsa_circ_0025039 (circFOXM1) were differential expressed (|Fold Change| > 2,*P* < 0.01). Only hsa_circ_0025039 (circFOXM1) was abundant in NSCLC cells (Fig.S6).

The ceRNA hypothesis proposes that RNA transcripts share the same miRNA response elements, resulting in competing for binding to miRNAs, then modulating the expression of each other [38]. In our study, we firstly combined the bioinformatic analyses and circFOXM1 pull-down assays to screen miRNAs, which bind with circFOXM1. Simultaneously, we designed the circFOXM1 luciferase reporter to identify the direct interaction between circFOXM1 and miRNA. We found that miR-614 was the most enriched miRNA in circFOXM1 pull-down assay, and miR-614 could reduce the luciferase activity of circFOXM1 luciferase reporter by about 50% (Fig. 4h). However, some miRNAs, such as miR-198, miR-516, and miR-6812, could also be enriched in H1299 cells, but not be enriched in H2170 cells. This observation suggests that the mechanism of circFOXM1 may be diverse in different cell lines.

We next analyzed the data of RNA-sequencing and results of miR-614 target prediction, and found FAM83D, a cell-cycle related gene, shared the same miR-614 response elements with circFOXM1. Based on the ceRNA hypothesis, FAM83D should be modulated by circFOXM1/miR-614 ceRNA regulatory network. After a series of experiments, such as RNA pull-down assay, luciferase assay, western blot assay, and qRT-PCR assay, we confirmed that FAM83D was indeed regulated by circFOXM1. Recently, similar mechanisms have been discovered, such as circMOT1/miR-9/p21 regulatory axis [39] and circNRIP1/miR-149/AKT regulatory axis [40].

FAM83D, a microtube associated protein, is reported frequently in various kinds of cancers as an oncogene. So far, FAM83D is considered to be associated with proliferation, migration, and invasion in cancer cells [41]. In NSCLC, it has been reported that FAM83D is a master cell-cycle regulator, resulting in CCND1 and CCNE1 alteration [16]. Although FAM83D function has been studied comprehensively, the regulators of FAM83D are largely unknown. Recently, several studies indicate that FAM83D can be regulated by miRNAs. For example, Yan et al. report that FAM83D is a direct target of miR-495 in colorectal cancer cells [14]. He et al. show that FAM83D expression is repressed by miR-210 during cell mitosis [15]. In our study, we first report that FAM83D is directly repressed by miR-614 and it is an essential partner of the circFOXM1 ceRNA regulatory network.

## Conclusions

**Fig. 7:**
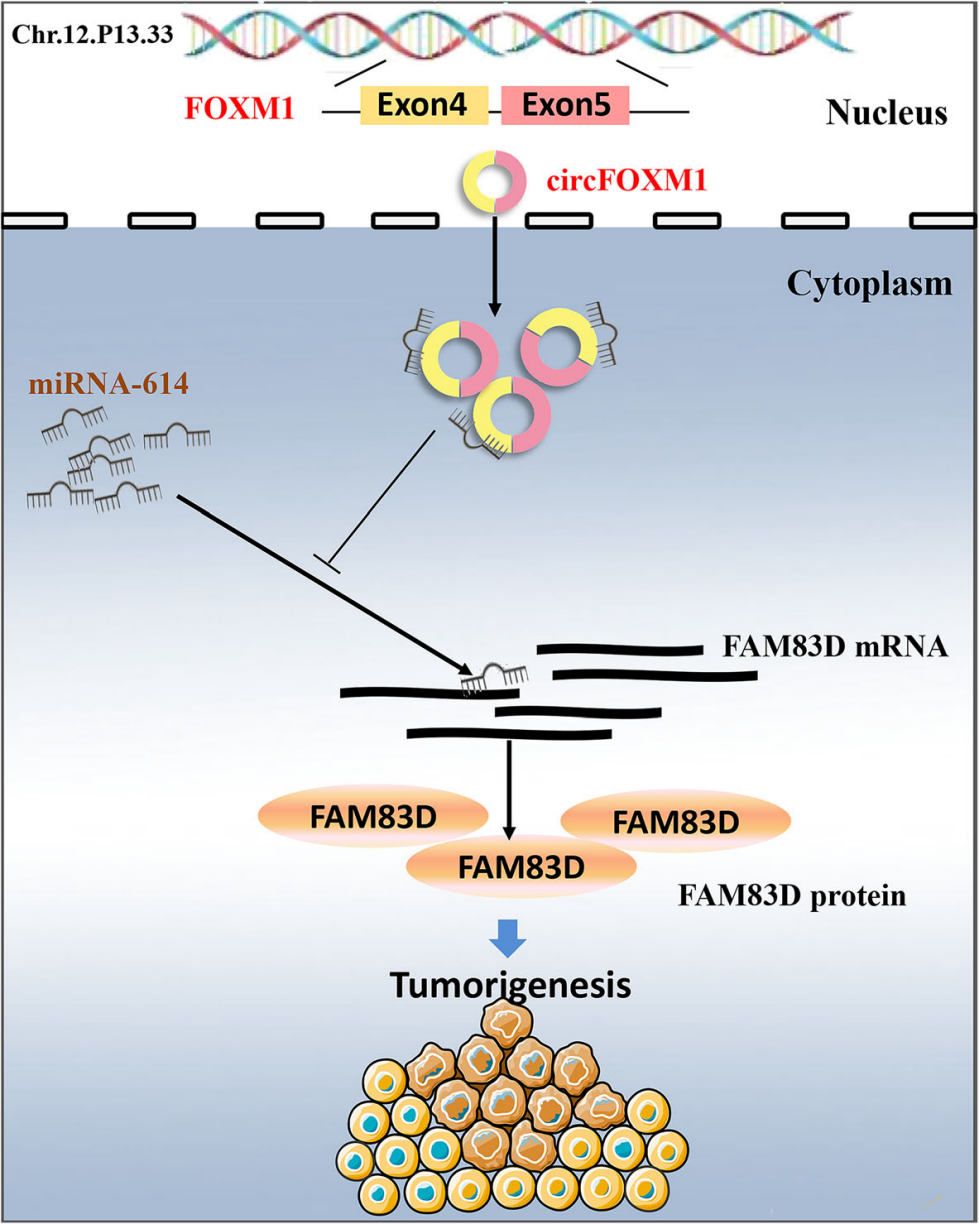
Schematic diagram of circFOXM1 effects on NSCLC cells proliferation. circFOXM1 acts as a sponge with miR-614 and releases its target FAM83D, resulting in the promoting effect on NSCLC cells proliferation

In summary, our study reveals that circFOXM1 competitively binds to miR-614 to abolish the suppressive effect of miR-614 on FAM83D, and thus promotes cell proliferation (Fig. 7). Our findings provide an insight into understanding the progression of NSCLC and a potential therapeutic biomarker for NSCLC.

## Supplementary information

**Additional file 1: Table S1.** The main primers used in this study.

**Additional file 2: Table S2.** The all 685 differently expressed genes in H1299 cells after silencing circFOXM1.

**Additional file 3: Table S3.** Predicted 54 mRNAs targeting by miR-614(8 mer sites).

**Additional file 4: Figure S1.** Overexpressing or silencing circFOXM1 could not disturb the expression of FOXM1. (A)Kaplan–Meier survival analysis of circFOXM1 expression in NSCLC patients. (B) Expression of circFOXM1 in1 normal cell line (a human bronchial epithelial cell line BEAS-2B) and 4 NSCLC cell lines. (C) Schematic view for construction of circFOXM1 overexpression vector. (D) Schematic view for sh-circFOXM1 targeted site. (E) mRNA levels of circFOXM1 and FOXM1 in H1299 and H2170 cells after transduction with circFOXM1 overexpression vector. (F) mRNA levels of circFOXM1 and FOXM1 in H1299 and H2170 cells after transduction with circFOXM1 shRNA. (G) Protein levels of FOXM1 in H1299 and H2170 cells with circFOXM1 overexpression. (H) Protein levels of FOXM1 in H1299 and H2170 cells with circFOXM1 knockdown. *P < 0.05; **P < 0.01.

**Additional file 5: Figure S2.** circFOXM1 mutation could not promote NSCLC cells proliferation and cell cycle progression. (A) Western blot analysis for Flag-Ago2 or Flag-tag expression. (B) CCK-8 assay was performed to determine the viability in H1299 and H2170 cells with overexpressing circFOXM1 mutation (circFOXM1 mut). (C) Flow cytometry analysis showed no obvious alteration in the proportion of cell in G1 phase and G2/M phase in H1299 and H2170 cells with overexpressing circFOXM1 mut.

**Additional file 6: Figure S3.** miR-614 inhibits cell proliferation. (A-B) CCK-8 and colony formation assays were performed to determine the viability in H1299 and H2170 cells after transfecting with miR-614 mimics. (C-D) Flow cytometry analysis showed increase in the proportion of cell in G1 phase and decrease in the proportion of cell in S and G2/M phase after transfecting with miR-614 mimics. (E) Expression of miR-614 was significantly downregulated in the 5 paired samples of NSCLC tissues by analysis in our GSE126533 data. T, tumor tissue; N, nontumor tissue. (F) Expression of miR-614 was examined in 20 paired samples of NSCLC. U6 was used as control. (G) Kaplan–Meier survival analysis of miR-614 expression in NSCLC patients by analysis of Kaplan Meier-plotter database. *P < 0.05; **P < 0.01.

**Additional file 7: Figure S4.** Silencing FAM83D inhibits cell proliferation. (A-B) CCK-8 and colony formation assays were performed to determine the viability in H1299 and H2170 cells with silencing FAM83D. (C-D) Flow cytometry analysis showed an alteration in the proportion of cell in G1 phase and G2/M phase in NSCLC cells with FAM83D silencing. (E) Expression of FAM83D was significantly upregulated in the 5 paired samples of NSCLC tissues by analysis in our GSE126533 data. (F) Expression analysis for FAM83D mRNA in TCGA database. (G) Kaplan-Meier survival analysis of FAM83D expression in NSCLC patients by analysis of Kaplan Meier-plotter database. (H) Correlation analysis of circFOXM1 and FAM83D in NSCLC tissues. *P < 0.05; **P < 0.01.

**Additional file 8: Figure S5.** Absolute quantification for circFOXM1 and FAM83D. Absolute quantification for circFOXM1 and FAM83D mRNA in H1299 and H2170 cells.

**Additional file 9: Figure S6.** Expression of circRNAs derived from FOXM1 in NSCLC cells. Relative expression of hsa_circ_0025039 (circFOXM1), hsa_circ_0025038, hsa_circ_0025042, hsa_circ_0025031, and hsa_circ_0025032 in NSCLC cells. **P < 0.01; ***P < 0.001.

## Data Availability

The datasets used and analyzed in the current study are available from the corresponding author on reasonable request.

## Abbreviations

NSCLC: Non-small cell lung cancer
circRNAs: Circular RNAs
miRNA: MicroRNA
FAM83D: Family with sequence similarity 83-member D
Cyclin D1: CCND1
Cyclin E1: CCNE1
CCK-8: Cell Counting Kit-8
FISH: Fluorescence in situ hybridization
