# Supplementary figures and images for: circFOXM1 promotes proliferation of non-small cell lung carcinoma cells by acting as a ceRNA to upregulate FAM83D
Source: J Exp Clin Cancer Res. 2020 Mar 30;39:55. doi: 10.1186/s13046-020-01555-5 (PMC7106704; doi:10.1186/s13046-020-01555-5)

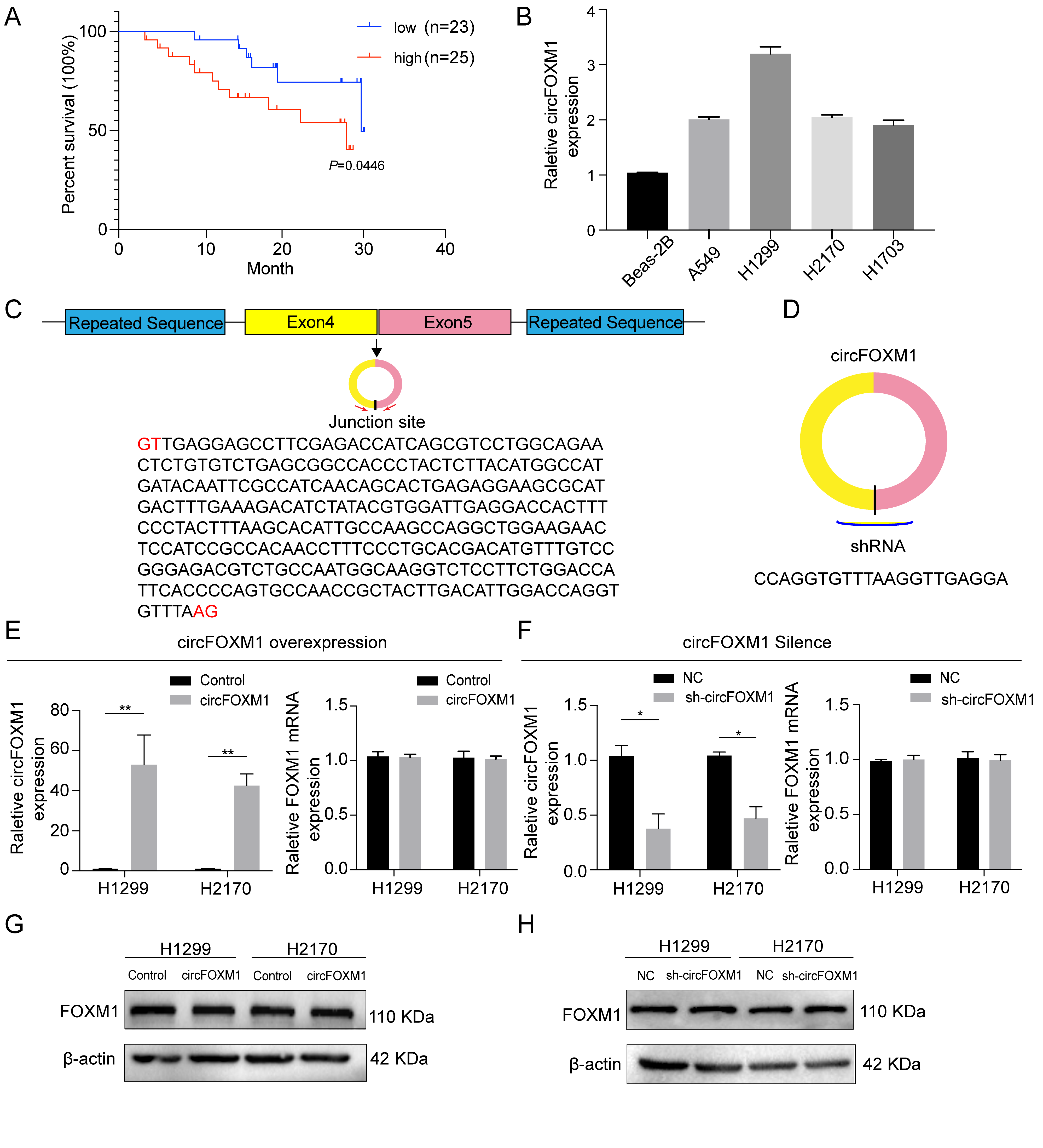

Supplement: Supplementary file 4 — Additional file 4: Figure S1. Overexpressing or silencing circFOXM1 could not disturb the expression of FOXM1. (A)Kaplan–Meier survival analysis of circFOXM1 expression in NSCLC patients. (B) Expression of circFOXM1 in1 normal cell line (a human bronchial epithelial cell line BEAS-2B) and 4 NSCLC cell lines. (C) Schematic view for construction of circFOXM1 overexpression vector. (D) Schematic view for sh-circFOXM1 targeted site. (E) mRNA levels of circFOXM1 and FOXM1 in H1299 and H2170 cells after transduction with circFOXM1 overexpression vector. (F) mRNA levels of circFOXM1 and FOXM1 in H1299 and H2170 cells after transduction with circFOXM1 shRNA. (G) Protein levels of FOXM1 in H1299 and H2170 cells with circFOXM1 overexpression. (H) Protein levels of FOXM1 in H1299 and H2170 cells with circFOXM1 knockdown. *P < 0.05; **P < 0.01. [file 13046_2020_1555_MOESM4_ESM.tif]

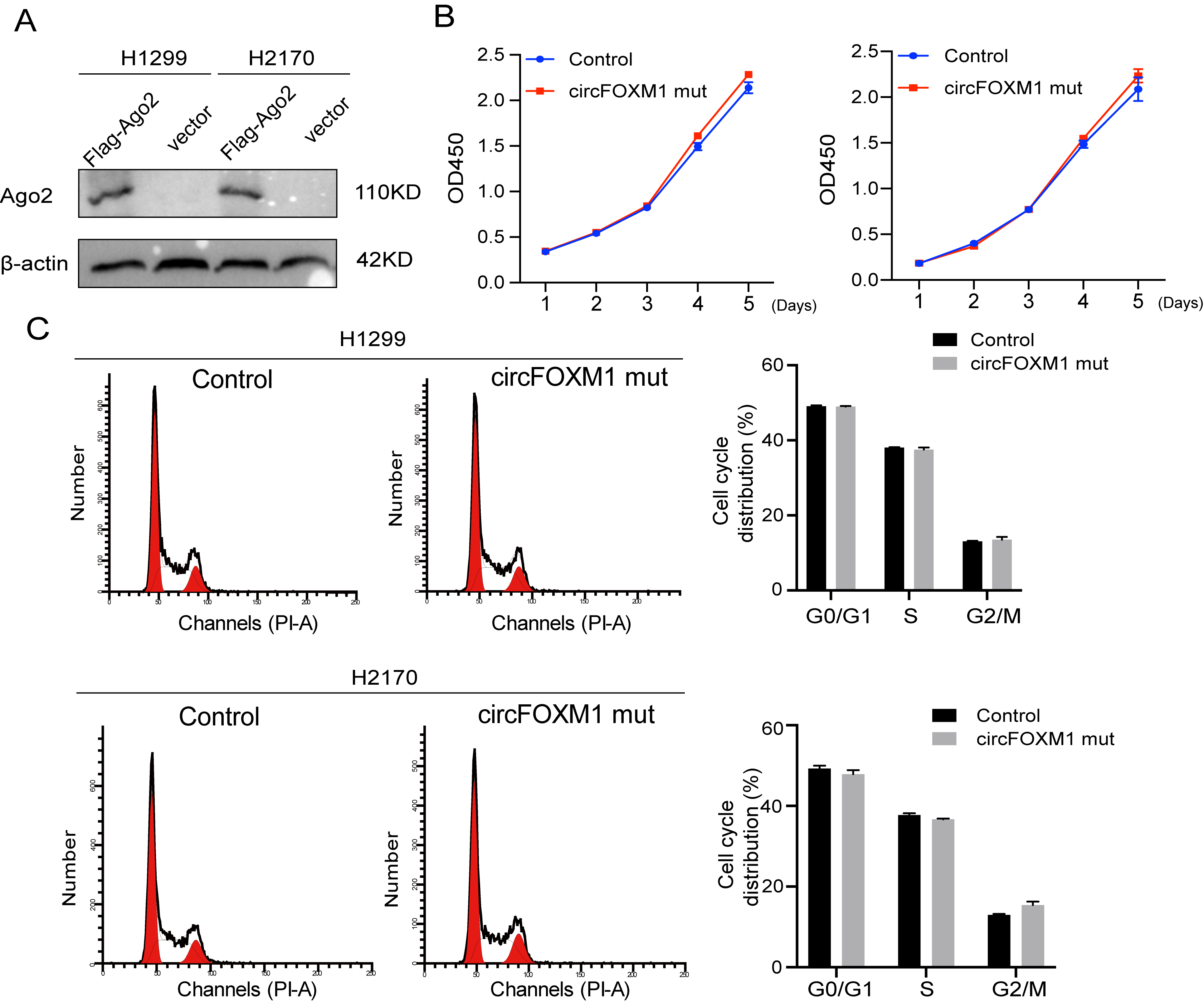

Supplement: Supplementary file 5 — Additional file 5: Figure S2. circFOXM1 mutation could not promote NSCLC cells proliferation and cell cycle progression. (A) Western blot analysis for Flag-Ago2 or Flag-tag expression. (B) CCK-8 assay was performed to determine the viability in H1299 and H2170 cells with overexpressing circFOXM1 mutation (circFOXM1 mut). (C) Flow cytometry analysis showed no obvious alteration in the proportion of cell in G1 phase and G2/M phase in H1299 and H2170 cells with overexpressing circFOXM1 mut. [file 13046_2020_1555_MOESM5_ESM.tif]

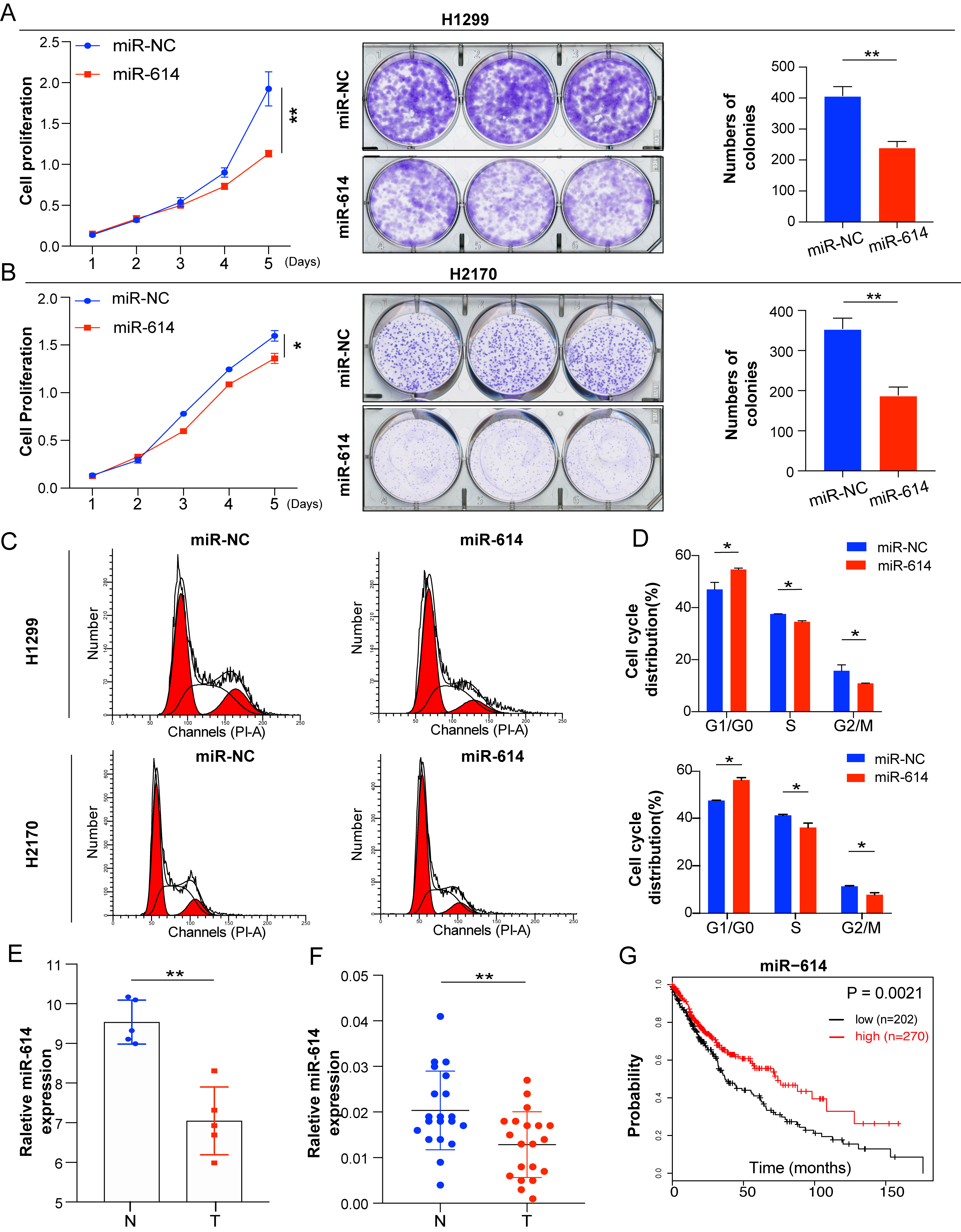

Supplement: Supplementary file 6 — Additional file 6: Figure S3. miR-614 inhibits cell proliferation. (A-B) CCK-8 and colony formation assays were performed to determine the viability in H1299 and H2170 cells after transfecting with miR-614 mimics. (C-D) Flow cytometry analysis showed increase in the proportion of cell in G1 phase and decrease in the proportion of cell in S and G2/M phase after transfecting with miR-614 mimics. (E) Expression of miR-614 was significantly downregulated in the 5 paired samples of NSCLC tissues by analysis in our GSE126533 data. T, tumor tissue; N, nontumor tissue. (F) Expression of miR-614 was examined in 20 paired samples of NSCLC. U6 was used as control. (G) Kaplan–Meier survival analysis of miR-614 expression in NSCLC patients by analysis of Kaplan Meier-plotter database. *P < 0.05; **P < 0.01. [file 13046_2020_1555_MOESM6_ESM.tif]

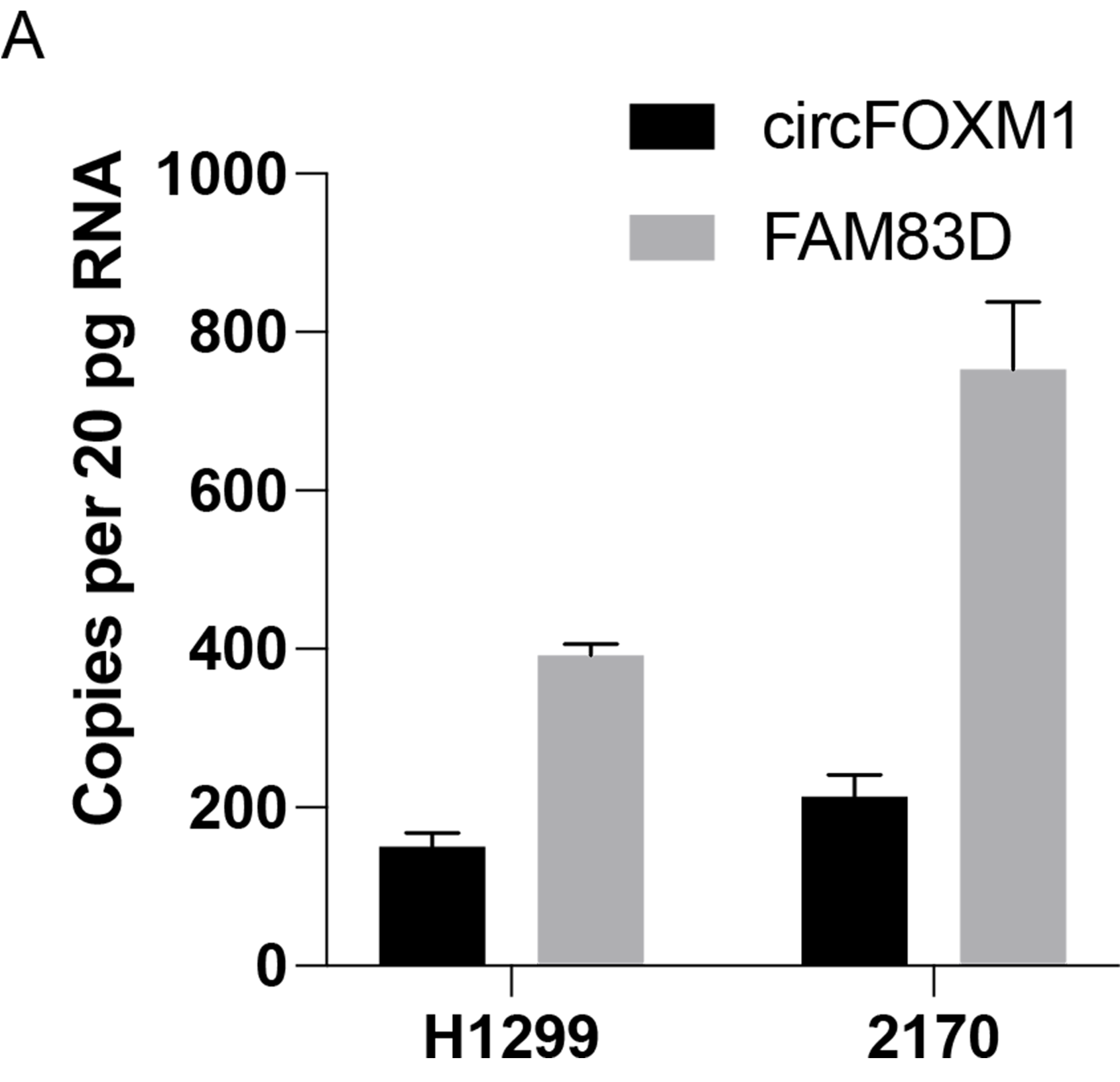

Supplement: Supplementary file 8 — Additional file 8: Figure S5. Absolute quantification for circFOXM1 and FAM83D. Absolute quantification for circFOXM1 and FAM83D mRNA in H1299 and H2170 cells. [file 13046_2020_1555_MOESM8_ESM.tif]

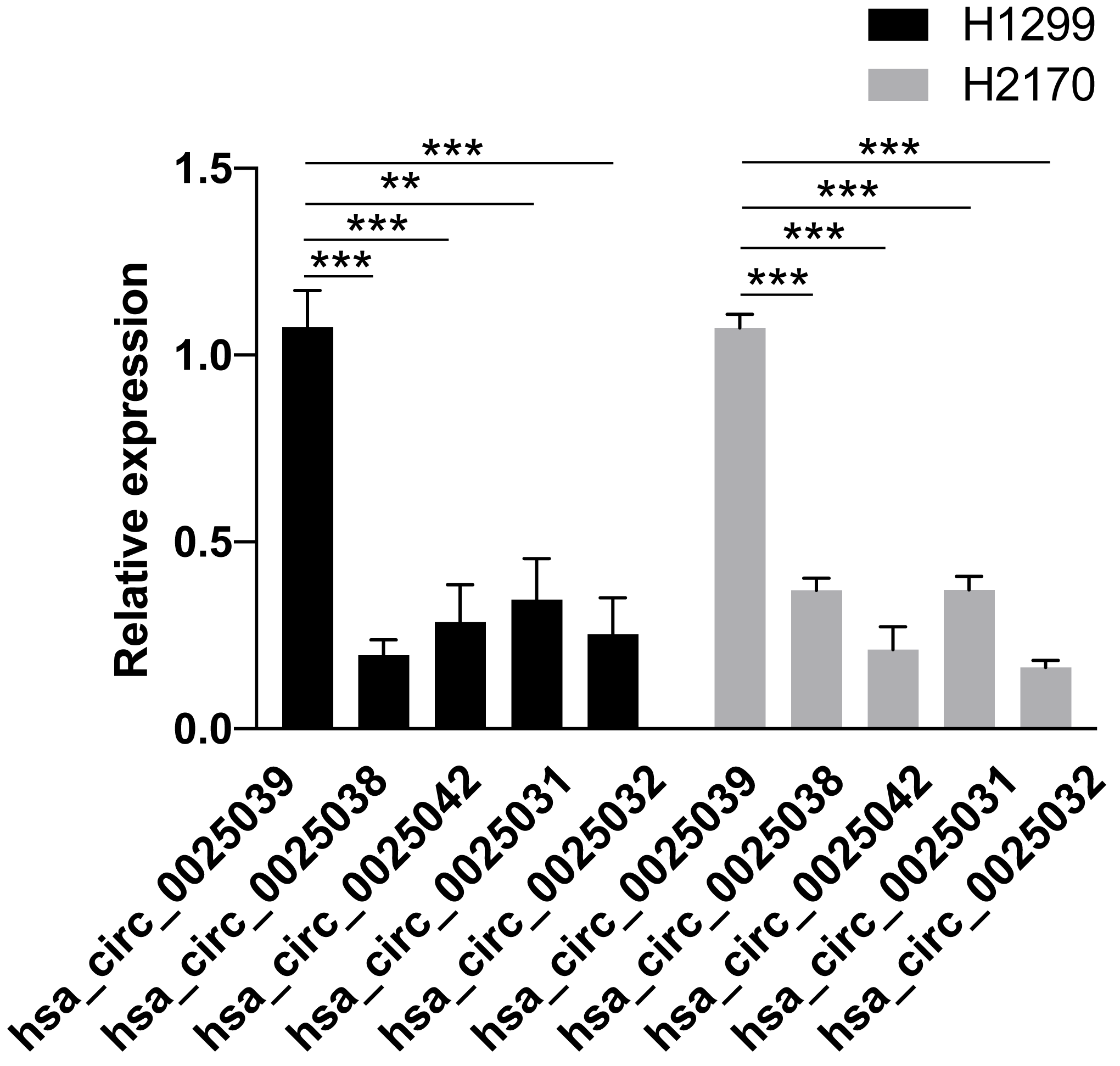

Supplement: Supplementary file 9 — Additional file 9: Figure S6. Expression of circRNAs derived from FOXM1 in NSCLC cells. Relative expression of hsa_circ_0025039 (circFOXM1), hsa_circ_0025038, hsa_circ_0025042, hsa_circ_0025031, and hsa_circ_0025032 in NSCLC cells. **P < 0.01; ***P < 0.001. [file 13046_2020_1555_MOESM9_ESM.tif]
